# P-1138. Quantifying Antibiotic Prescribing in Children with Tracheostomies

**DOI:** 10.1093/ofid/ofae631.1325

**Published:** 2025-01-29

**Authors:** Rebecca Steuart, Austin Slone, Joshua D Courter, Dan Benscoter, Amy Pan, Samir S Shah, Joanna Thomson

**Affiliations:** Medical College of Wisconsin, Milwaukee, Wisconsin; University of Cincinnati, Cincinnati, Ohio; Cincinnati Children's Hospital Medical Center, Cincinnati, Ohio; Cincinnati Children's Hospital Medical Center, Cincinnati, Ohio; Medical College of Wisconsin, MIlwaukee, Wisconsin; Cincinnati Children's Hospital Medical Center, Cincinnati, Ohio; Cincinnati Children's Hospital Medical Center, Cincinnati, Ohio

## Abstract

**Background:**

Prolonged or unnecessary antibiotic prescribing is associated with adverse outcomes. Children with tracheostomies are at risk for antibiotic overprescribing. We sought to quantify and characterize systemic antibiotic prescribing among children with tracheostomies, and to identify predictors of higher prescribing.

**Figure:**
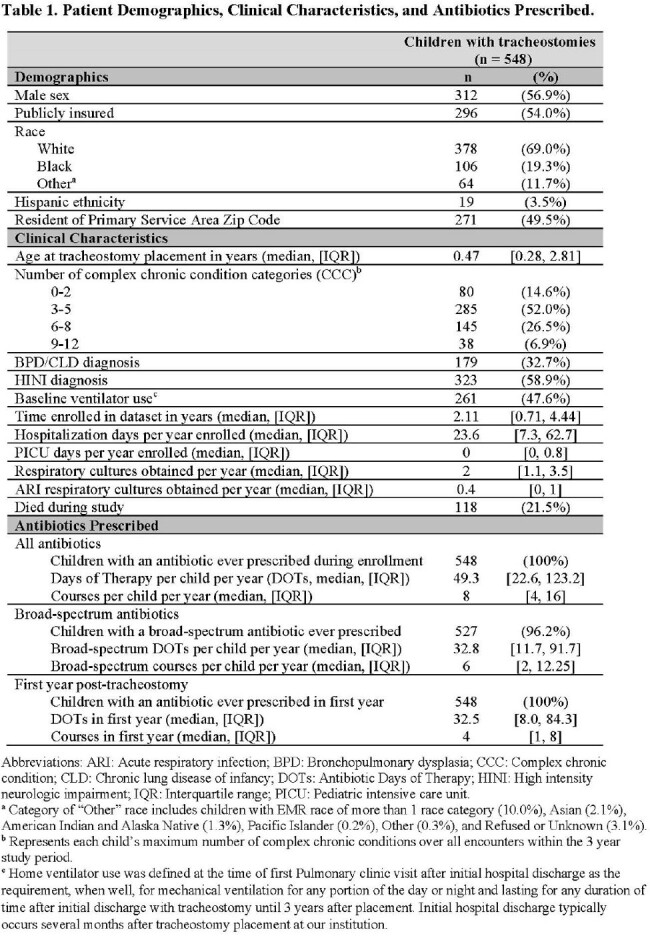

**Methods:**

This single-center retrospective cohort study included children with tracheostomies 2 months-18 years old cared for between 2010-2018. Detailed demographic, clinical, and antibiotic prescribing data were extracted from the medical record. Patient-level antibiotic prescribing was quantified using antibiotic courses initiated and DOT per child per year of enrollment. Group-level prescribing was summarized using total antibiotic courses and days of therapy (DOT) per 1000 person-days. Antibiotics prescribed for > 1 day were classified by spectrum of activity and stratified by setting and year. Clinical predictors of patient-level prescribing were identified using backward stepwise multivariable analyses within a generalized linear model.

**Figure:**
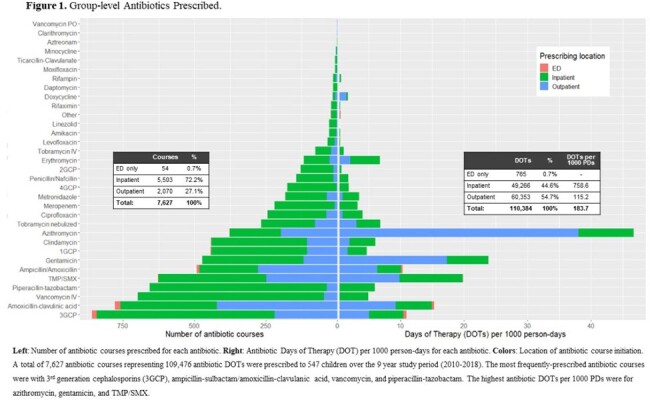

**Results:**

The 548 children with tracheostomies (47.6% with baseline ventilator use) were prescribed a median 8 antibiotic courses per child per year (IQR: 4-16) for a total median exposure of 49.3 DOT per child per year (IQR: 22.6-123.2) or 183.7 DOTs per 1000 person-days. Most courses (73.9%) were broad-spectrum, with vancomycin, piperacillin-tazobactam, and ampicillin-clavulanate being the top 3 most frequently-prescribed antibiotic courses. Azithromycin and gentamicin represented a high proportion of all DOTs (azithromycin 25.4%, gentamicin 13.0%). Annual antibiotic prescribing decreased 34.5% from 2010-2018. In adjusted analysis to identify predictors, ventilator use at baseline was associated with fewer DOT per child per year, while higher patient complexity and more ICU hospital days were risk factors for higher DOT.

**Figure:**
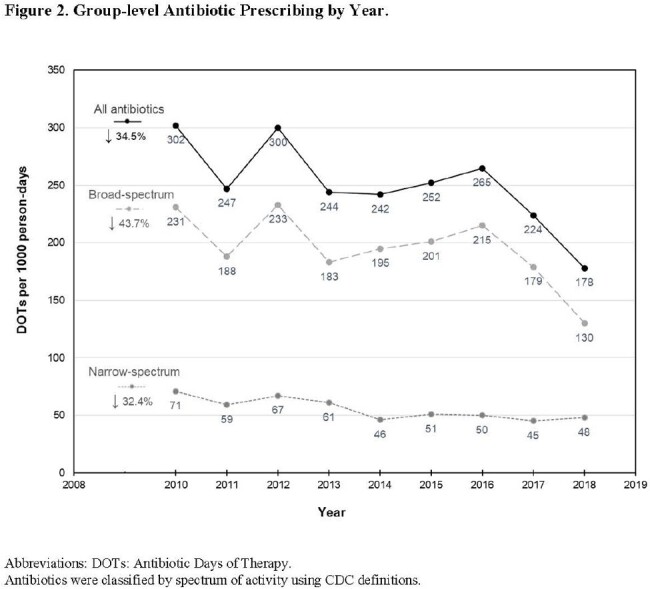

**Conclusion:**

Children with tracheostomies have high antibiotic use, and are predominantly prescribed broad-spectrum antibiotics. Chronic ventilator use was a protective factor against higher prescribing. This data may inform antimicrobial stewardship work.

**Figure:**
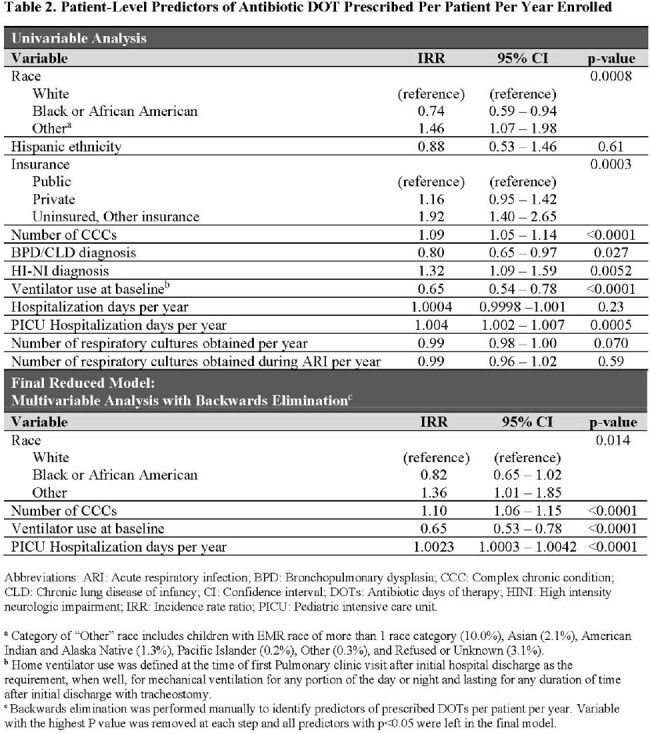

**Disclosures:**

**All Authors**: No reported disclosures

